# Epithelial Ablation of Miro1/Rhot1 GTPase Augments Lung Inflammation by Cigarette Smoke

**DOI:** 10.3390/pathophysiology28040033

**Published:** 2021-11-26

**Authors:** Shikha Sharma, Qixin Wang, Thivanka Muthumalage, Irfan Rahman

**Affiliations:** Department of Environmental Medicine, University of Rochester Medical Center, Rochester, NY 14642, USA; Shikha_Sharma@urmc.rochester.edu (S.S.); Qixin_Wang@urmc.rochester.edu (Q.W.); Thivanka_Muthumalage@urmc.rochester.edu (T.M.)

**Keywords:** Miro1, lung inflammation, mitochondrial quality control, cigarette smoke, COPD

## Abstract

Mitochondrial quality control is sustained by Miro1 (Rhot1), a calcium-binding membrane-anchored GTPase during mitophagy. The exact mechanism that operates the interaction of Miro1 with mitophagy machinery and their role in cigarette smoke (CS)-induced mitochondrial dysfunction that often results in lung inflammation is unclear. We hypothesized that Miro1 plays an important role in regulating mitophagy machinery and the resulting lung inflammation by CS exposure to mice. The lung epithelial Rhot1^fl/fl^ (WT) and Rhot1^CreCC10^ mice were exposed to mainstream CS for 3 days (acute) and 4 months (chronic). Acute CS exposure showed a notable increase in the total inflammatory cells, macrophages, and neutrophils that are associated with inflammatory mediators. Chronic exposure showed increased infiltration of neutrophils versus air controls. The effects of acute and chronic CS exposure were augmented in the Rhot1^CreCC10^ group, indicating that epithelial Miro1 ablation led to the augmentation of inflammatory cell infiltration with alteration in the inflammatory mediators. Thus, Rhot1/Miro1 plays an important role in regulating CS-induced lung inflammatory responses with implications in mitochondrial quality control.

## 1. Introduction

Cigarette smoke (CS) leads to mitochondrial dysfunction that is associated with lung inflammation [1] Mitochondrial dysfunction accelerates the inflammatory process in lung diseases such as chronic obstructive pulmonary disease (COPD) [2]. Normal cellular homeostasis and physiology depend on the regulation of mitochondrial function [3]. During cellular damage, dysfunctional mitochondria are eliminated through a selective degradation process called mitophagy [4]. To sustain the quality control of the mitochondria, Miro1 (Rhot1) serves as an essential element in mitochondrial quality control. Rhot1 (Miro1) is a calcium-binding membrane-anchored GTPase that is required for the calcium-driven movement of the mitochondria in microtubules, especially during mitophagy [1,5,6,7]. Miro1 also serves a vital role in mitochondrial dynamics because of its interaction with the Pink1 (PTEN-induced putative kinase 1)/Parkin mitochondrial quality control system by serving as a signal for mitophagy [8,9]. The depolarization of the mitochondrial membrane is responsible for initiating mitophagy in the cells. Following this, mitochondrial quality control is performed by the stabilization of Pink1 on the outer membranes of the mitochondria through fission reactions. An E3 ubiquitin ligase, also called Parkin, is recruited from the cytosol by the Pink1 for further mitochondrial quality control [1,10]. After the recruitment of Parkin, the degradation of a mitochondrial fusion core protein—mitofusin2 (Mfn2) occurs. This allows the damaged mitochondria to be cleared from the cell by protein microtubule-associated protein light chain 3 (LC3) in the isolation membranes. This phenomenon further initiates the formation of the autophagosome by the localization of damaged mitochondria that, in turn, fuse with the lysosomes to remove the dead mitochondrial cells [11,12]. Pink1 and Parkin thereby coordinate together during mitophagy to help regulate mitochondrial degradation. Mitochondrial shape and trafficking are maintained by GTPase Miro1 and Miro2. Thus, together, they may serve a crucial role in mitochondrial quality control [13]. However, the exact mechanism that operates this interaction and the role of Miro1 in CS-induced mitochondrial dysfunction that results in lung inflammation in COPD remains unclear.

We hypothesize that the ablation of Miro1 (Rhot1) in lung epithelial cells would result in the dysfunction of mitochondrial quality control (dysfunctional mitophagy). This may, in turn, exaggerate the adverse effects of cigarette smoke in the lungs, thereby ensuing CS-induced lung inflammation [1,7]. To determine the role of Rhot1/Miro1 in augmenting the CS-induced lung inflammation and the effect of duration of exposure on its implication on COPD/emphysema phenotype development, we utilized epithelial cell-specific (Rhot1^CreCC10^) Miro1 knockout mouse models that were exposed to mainstream CS for 3 days (acute phase or short term) and 4 months (chronic phase or long term). The role of mitochondrial Miro1 and its impact on the progression of lung inflammation by acute and chronic CS exposure in mice was determined.

## 2. Materials and Methods

The study was approved via the laboratory protocols of the Institutional Biosafety Committee (IBC) of the University of Rochester Medical Center, Rochester, NY, USA. All of the animal experiments were approved by the University Committee on Animal Research at the University of Rochester, Protocol no.102204/2007-070E, date of approval, 31 January 2019.

### 2.1. Animal Model and Exposure

Adult male and female wild-type (Rhot1^fl/fl^) and lung epithelial cell-specific Rhot1 KO (Rhot1^CreCC10^) mice were exposed to 3-day acute and 4-month chronic mainstream cigarette smoke. Rhot1flpflox CreCC10 and Rhot1flox CreCC10 strains (hereinafter collectively referred to as Rhot1 ^CreCC10^) were used for 3-days and 4-months, respectively.

The mice Rhot1flp were purchased from MMRRC, CA, USA and Rhot1flox were purchased from Jackson Laboratory, Bar Harbor, ME, USA. Both flp and flox techniques are based on the LoxP/Cre-mediated generation of conditional gene KO. The KO group is commonly denoted as Rhot1^CreCC10^. Air group mice were exposed to filtered room air. Animals were 2–4 months old at the start of the exposures and were housed under standard pathogen-free conditions with a 12/12 h light and dark cycle in the University of Rochester Medical Center vivarium facility.

### 2.2. Cigarette Smoke Exposure

Cigarette smoke for exposure was generated by using 3R4F (research-grade cigarettes), and the exposure guidelines as per the protocol of the Federal Trade Commission (1 puff/minute of 2-s duration with the volume of 35 mL) were followed for 3 days for acute exposures and for 4 months for chronic exposures, the mice being exposed to CS 5 days/week, with 2 h of exposure per day using a Baumgartner-Jaeger CSM2082i (cigarette smoke generating machine; CH Technologies, Westwood, NJ, USA) [14,15,16]. The mainstream smoke concentration was set at ~250 mg/m^3^ of the TPM value after diluting it with filtered air, and the concentration of carbon monoxide (290 to 300 ppm) was monitored in the chamber [17,18,19]. Simultaneously, all of the air group mice were exposed to normal filtered room air at the same duration as those exposed to CS groups [15,20].

### 2.3. Collection of Bronchoalveolar Lavage (BAL)

The mice were injected with 100 mg/kg (BW) of pentobarbital sodium (Abbott Laboratories, Green Oakes, IL, USA) intraperitoneally and were then euthanized. Then, a cannula was inserted into the trachea of the mice, and the lungs were lavaged with normal saline (0.6 mL volume; 3 times). The lavage fluid was collected and was centrifuged, and the separated supernatant samples were stored at −80 °C [20,21,22,23].

### 2.4. Total Cell Count in BAL Fluid

The cell pellet of BAL fluid was resuspended in normal saline (0.9% NaCl; 1 mL/vol.) and was stained by trypan blue (Cat# 15250061, Thermo Fisher Scientific, Waltham, MA, USA), and the total cell count/mL was determined by TC-20 Automated Cell Counter (BioRad, Hercules, CA, USA).

### 2.5. Differential Cell Count in BAL Fluid

For 3 days (acute) of exposure, the differential cell count of immune-inflammatory cells (neutrophils, macrophages, CD4 T-lymphocytes, CD8 T-lymphocytes) was completed via Guava^®^ easyCyte™ flow cytometer (Millipore Sigma, Burlington, MA, USA) in BAL fluid. All of the cells were stained using cell type-specific mAb. The specific cell labeling markers LY6B.2 Alexa Fluor 488 (Cat#NBP213077AF488, Novus Biologicals, Littleton, CO, USA), F4/80 phycoerythrin (Cat#123109, BioLegend, San Diego, CA), CD45 allophycocyanin (Cat#110728, BioLegend, San Diego, CA), CD4a PE-Cy7 (Cat#25–0041-82, Fisher Scientific, Waltham, MA), and CD8a phycoerythrin-Cy5 (Cat#17–0081-82, Fisher Scientific, Waltham, MA) were used for neutrophils, macrophages, leukocytes, and T-lymphocytes, respectively.

For four months (chronic) of exposure, differential cell counts were performed to determine the cell influx of neutrophils and macrophages on cytospin slides with Diff-Quick staining [24,25]. Diff-Quick staining involves the sequential dipping of the slides into different solutions—fixative agent (methanol, blue), solution 1 (eosinophilic, orange), and solution 2 (basophilic, blue), followed by rinsing and drying. The collected smears were first allowed to dry, and the slides were then dipped for one second, which was repeated five times each into a fixative, followed by stains 1 and 2. The excess was allowed to drain after each dip. After drying, the slide was rinsed with Weise’s buffer (pH 7.2). It was then blotted or was allowed to dry in the air for further examination.

### 2.6. Inflammatory Mediators

The levels of inflammatory mediators in BAL fluid were assessed by the Bio-Plex Pro Mouse Cytokine 23-Plex Immunoassay (Cat# M60009RDPD, BioRad, Hercules, CA, USA) via Luminex. Experiments were completed following the manufacturer’s instructions, and the results were presented as pg/mL in the samples.

### 2.7. Measurement of Lung Mechanics

The parameters of the lung mechanics, such as lung resistance, tissue elastance, and static compliance, were measured by Flexivent apparatus (Scireq; Montreal, QC, Canada). All of the mice were anesthetized using pentobarbital (90 mg/kg; i.p. injection). Mice were then tracheostomized and cannulated, and the cannula was attached to the rodent ventilator, utilizing a computer [18,26]. Measurements of lung mechanical properties were repeated three times.

### 2.8. Lung Morphometry and Histopathology

Mouse lungs were isolated and inflated with 1% agarose (low melting) and were fixed with 4% neutral-buffered paraformaldehyde [27]. The tissues were dehydrated and embedded in paraffin for sectioning using a rotary microtome. The midsagittal lung section of each tissue sample was stained with H&E (hematoxylin and eosin), and the Lm (linear intercept) of the airspace and histopathological changes were determined (20×) using Metamorph software (Molecular device, San Jose, CA, USA).

### 2.9. Statistical Analysis

Statistical analysis was completed with one-way (ANOVA) and Tukey’s post hoc multiple group comparison test using GraphPad Prism 9 software (version 9, GraphPad, San Diego, CA, USA). Data are shown as mean ± SEM. Significance was compared between corresponding Air and CS groups of the same genotype as well as in different genotypes. *p* < 0.05 is considered significant.

## 3. Results

CS-induced inflammatory cell infiltration and the differential expression of cytokines were observed in lung epithelial cell-specific Rhot1-deleted mice after 3 days of acute and 4 months of chronic CS exposures.

### 3.1. Three Days (Acute) Exposure

#### 3.1.1. Inflammatory Cellular Influx in Rhot1 Epithelial Cell Specific KO (Rhot1^CreCC10^) and WT (Rhot1^fl/fl^) Mice

**Figure 1:**
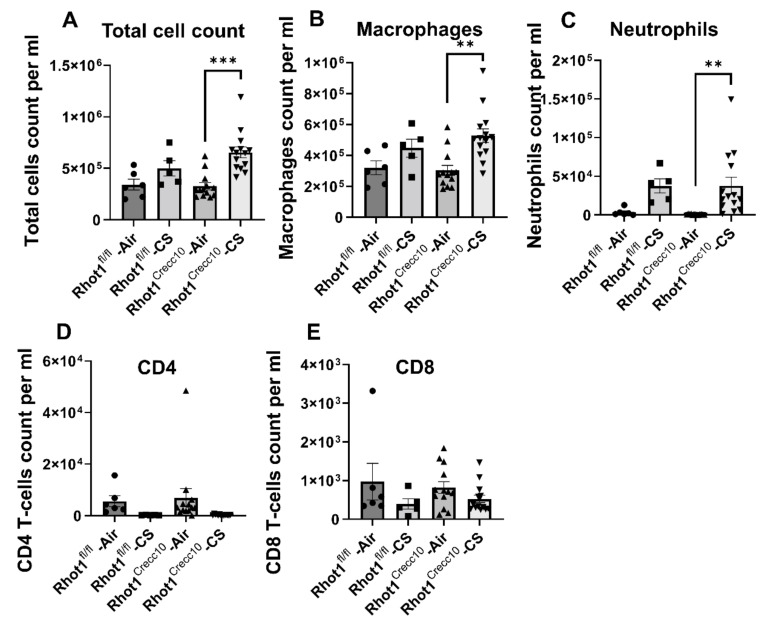
CS-induced cell infiltration in lung epithelial cell-specific Rhot1-deleted mice: Rhot1^fl/fl^ (WT) and Rhot1^CreCC10^ (Rhot1 flp ^CreCC10+/−^ and Rhot1 flp ^CreCC10+/+)^ mice were exposed to room air and CS (mainstream) for 3 days (acute exposure). Differential cell counts in the BAL fluid of filtered air-exposed mice and in mice that were exposed to CS for 3 days were determined. Alteration in (**A**). Total cell count, (**B**). Macrophage, (**C**). Neutrophils, (**D**). CD4 T-cells and (**E**). CD8 T-cells occurred due to CS exposure. Significance compared between corresponding Air and CS groups and all of the groups compared with each other irrespective of their exposure. The symbols on the bar graphs are representing individual groups/data: circles-Rhot1^fl/fl^—Air; squares-Rhot1^fl/fl^—CS; upward triangles-Rhot1^CreCC10^—Air; downward triangles-Rhot1^CreCC10^—CS. Data are shown as mean ± SEM (*n* = 5 to 13 per group). ** *p* < 0.01, *** *p* < 0.001.

The total number of cells and the macrophage and neutrophil counts only increased in the Rhot1 epithelial cell-specific KO (Rhot1^CreCC10^) mice exposed to CS for 3 days, whereas upregulation trends were observed in the WT (Rhot1^fl/fl^) mice exposed to CS, but these trends were not significant. However, none of the groups showed significant changes in the levels of CD4 and CD8 T-lymphocytes in response to CS exposure (Figure 1).

#### 3.1.2. Effect of Acute, CS Exposure on Levels of Pro-Inflammatory Mediators in Rhot1 Epithelial Cell Specific KO (Rhot1^CreCC10^) and WT (Rhot1^fl/fl^) Mice

**Figure 2:**
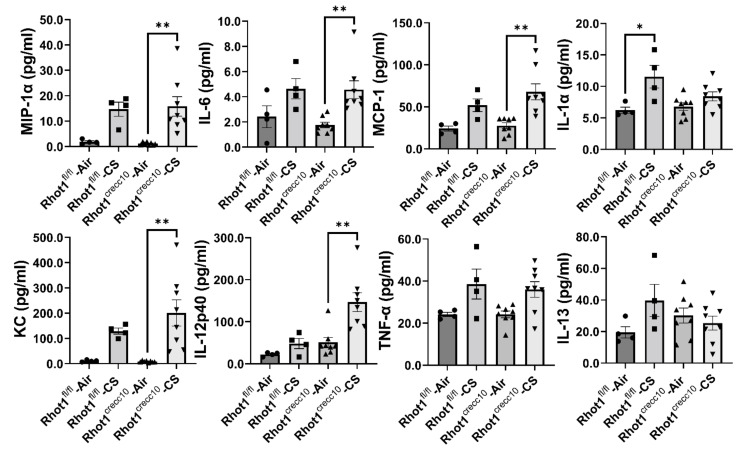
Differential expression of cytokines in epithelial cell-specific-Rhot1 deleted and WT mice: Rhot1^fl/fl^ (WT) and Rhot1^CreCC10^ (Rhot1 flp ^CreCC10+/−^ and Rhot1 flp ^CreCC10+/+^) mice were exposed to room air and CS (mainstream) for 3 days (acute exposure). Expression levels of pro-inflammatory and inflammatory mediators in the BAL fluid from filtered air-exposed mice and mice that were exposed to CS for 3 days were determined using the Bio-Plex Pro 23-plex cytokine assay. Significance compared between corresponding Air and CS groups and all of the groups compared with each other irrespective of their exposures. The symbols on the bar graphs are representing individual groups: circles-Rhot1^fl/fl^—Air; squares-Rhot1^fl/fl^—CS; upward triangles-Rhot1^CreCC10^—Air; downward triangles-Rhot1^CreCC10^—CS. Data are shown as mean ± SEM (*n* = 5 to 8 per group). * *p* < 0.05, ** *p* < 0.01.

WT (Rhot1^fl/fl^) mice exposed to 3 days of CS showed increasing trends of several pro-inflammatory mediators, but the changes between the air and CS group were not significant except for IL-1α. CS-exposed Rhot1 epithelial cell-specific KO (Rhot1^CreCC10^) mice showed a significant increase in the levels of MCP-1, IL-6, IL-1α, IL-12p40, KC and MIP-1α, whereas TNF-α and IL-13 showed a trend in increased levels by CS (Figure 2). Interestingly, a significant increase in the levels of IL-1α with an increasing trend in other mediators MIP-1α, IL-6, MCP-1, KC, TNF-α, IL-13, and IFN-γ were observed in WT (Rhot1^fl/fl^) CS groups. The rest of the analyzed mediators were not significantly affected (Figure S1).

### 3.2. Four Months (Chronic) Exposure

#### 3.2.1. Inflammatory Cellular Influx in Rhot1 Epithelial Cell Specific KO (Rhot1^CreCC10^) and WT (Rhot1^fl/fl^) Mice by Chronic CS Exposures

**Figure 3:**
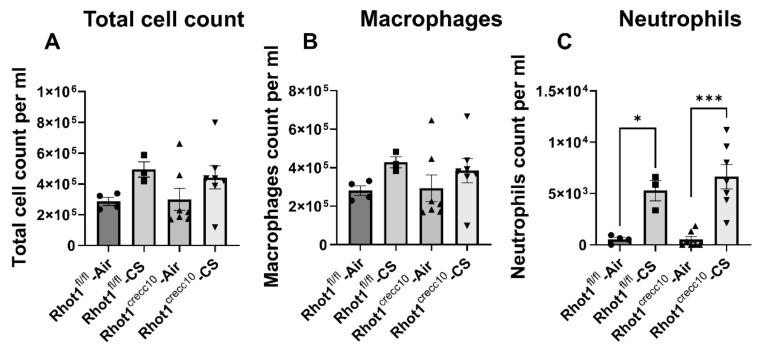
Increased neutrophil influx in Rhot1^CreCC10^ (Rhot1 Flox ^CreCC10+/−)^ mice in response to chronic CS exposure for 4 months. (**A**) The number of total cells in BAL fluid from air- and CS-exposed mice for 4 months was determined. (**B**) Lavaged macrophage and (**C**) neutrophil numbers were counted in Diff-Quik stained cytospin slides, which were prepared using BAL fluid. Quantification of macrophages and neutrophils expressed as absolute cell count per ml in BAL fluid from air- and CS-exposed mice. CS-exposed Rhot1^CreCC10^ (Rhot1 ^CreCC10+/−)^ mice showed a significant increase in total cell count and number of neutrophils. The symbols on the bar graphs are representing individual groups: circles-Rhot1^fl/fl^—Air; squares-Rhot1^fl/fl^—CS; upward triangles-Rhot1^CreCC10^—Air; downward triangles-Rhot1^CreCC10^—CS. Data are shown as mean ± SEM (*n* = 4 to 7 per group). * *p* < 0.05, *** *p* < 0.001 are significant compared to corresponding air-exposed mice.

Chronic CS exposure significantly increased the number of neutrophils in Rhot1^CreCC10^ mice, as well as in lymphocytes (data not shown). A non-significant but increasing trend was observed in the number of macrophages in the CS-exposed groups (Figure 3).

#### 3.2.2. Effect of Chronic, CS Exposure on Levels of Pro-Inflammatory Mediators in Rhot1 Epithelial Cell Specific KO (Rhot1^CreCC10^) and WT (Rhot1^fl/fl^) Mice

**Figure 4:**
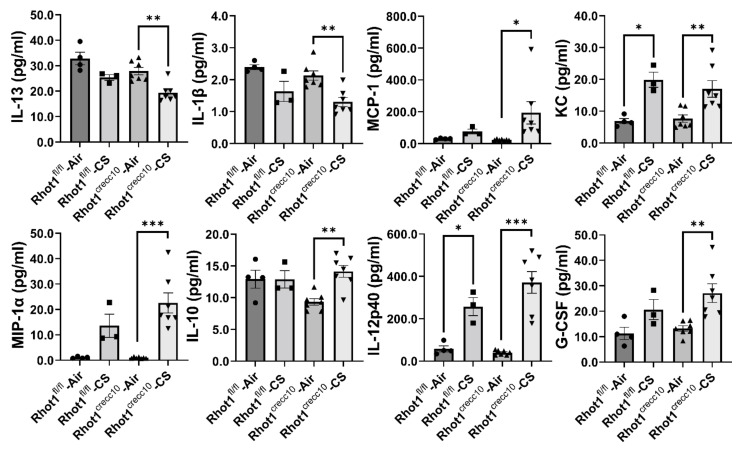
Differential expression of cytokines in epithelial cell-specific Rhot1-deleted and WT mice: Rhot1^fl/fl^ (WT) and Rhot1^CreCC10^ (Rhot1 flp ^CreCC10+/−^ and Rhot1 flp ^CreCC10+/+^) mice were exposed to room air and CS (mainstream) for 4 months (chronic exposure). Expression levels of pro-inflammatory and inflammatory mediators in the BAL fluid filtered air- and CS-exposed mice after 4 months, as determined using the Bio-Plex Pro 23-plex cytokine assay. Significance compared between corresponding Air and CS groups and all of the groups compared to each other irrespective of their exposures. The symbols on the bar graphs are representing individual groups: circles-Rhot1^fl/fl^—Air; squares-Rhot1^fl/fl^—CS; upward triangles-Rhot1^CreCC10^—Air; downward triangles-Rhot1^CreCC10^—CS. Data are shown as mean ± SEM (*n* = 3 to 7 per group). * *p* < 0.05, ** *p* < 0.01, *** *p* < 0.001 between groups.

CS-exposed WT (Rhot1^fl/fl^) and Rhot1 epithelial cell-specific KO (Rhot1^CreCC10^) mice showed significantly increased cytokine levels (KC andIL-12p40 compared to respective air groups (Figure 4). Intriguingly, the levels of MCP-1, MIP-1α, G-CSF, and IL-12p40 in BALF were more augmented in Rhot1 epithelial cell-specific KO (Rhot1^CreCC10^) mice than they were in WT (Rhot1^fl/fl^) mice by CS. However, the levels of G-CSF, MIP-1α, IL-10 and MCP-1 only showed a significant increase in Rhot1 epithelial cell-specific KO (Rhot1^CreCC10^) mice, and a slight increase in the levels of G-CSF, MIP-1α, and Eotaxin in Rhot1^fl/fl^ mice compared with respective WT air group. The rest of the mediators were not significantly affected (Figure 4 and Figure S2).

#### 3.2.3. Effect of Chronic CS on Lung Mechanical Properties in Rhot1 Epithelial Cell Specific KO (Rhot1^CreCC10^) WT (Rhot1^fl/fl^) Mice

**Figure 5:**
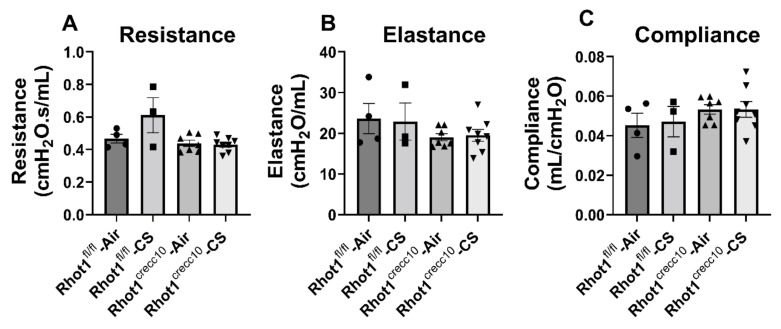
Alterations in lung mechanical properties in Rhot1^CreCC10^ (Rhot1 Flox ^CreCC10+/−^ mice in response to CS exposure for 4 months. (**A**) Tissue resistance, (**B**) elastance, and (**C**) static compliance were measured by FlexiVent after 4 months of air and CS exposure. Significance compared between corresponding Air and CS groups and all of the groups compared with each other irrespective of their exposures; none of the changes found were significant. The symbols on the bar graphs are representing individual groups: circles-Rhot1^fl/fl^—Air; squares-Rhot1^fl/fl^—CS; upward triangles-Rhot1^CreCC10^—Air; downward triangles-Rhot1^CreCC10^—CS. Data are shown as mean ± SEM (*n* = 3 to 8 per group).

WT (Rhot1^fl/fl^) and KO (Rhot1^CreCC10^) mice exposed to CS showed no significant changes in t mechanical property parameters, such as resistance, compliance, and elastance (Figure 5).

#### 3.2.4. Effect on Airspace Enlargement and Histopathology of Lung Tissues in Rhot1 Epithelial Cell Specific KO (Rhot1^CreCC10^) and WT (Rhot1^fl/fl^) Mice

**Figure 6:**
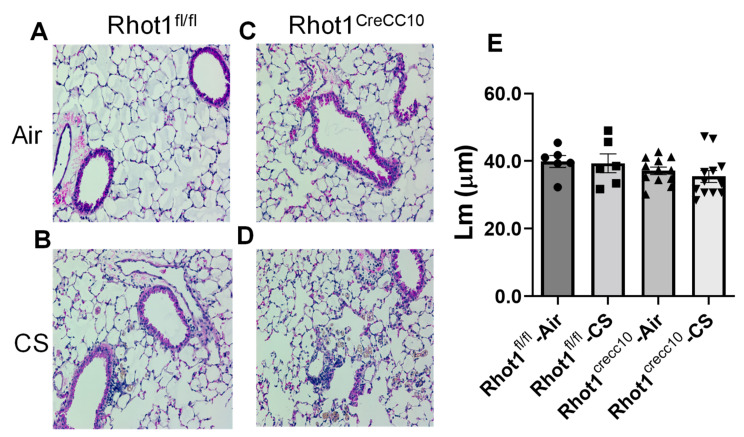
CS-induced alterations in airspace enlargement and changes in histopathology in lungs of Rhot1^CreCC10^ (Rhot1 Flox ^CreCC10+/−^ and Rhot1 Flox ^CreCC10+/−)^ mice exposed to CS for 4 months. The pictures shown are H&E-stained lung sections from air- and CS-exposed Rhot1^fl/fl^ (WT) and Rhot1^CreCC10^ (Rhot1 Flox CreCC10^+/−^ Rhot1 Flox CreCC10^+/−^) mice for 4 months. (**A**). Rhot1^fl/fl^—Air; (**B**). Rhot1^fl/fl^—CS; (**C**). Rhot1^CreCC10^—Air; (**D**). Rhot1^CreCC10^—CS. (**E**). Mean linear intercept (Lm) was measured in H&E-stained lung sections. Lung sections from air- and CS-exposed Rhot1 Flox CreCC10^+/−^ mice did not show significant airspace enlargement compared to air- and CS-exposed Rhot1^fl/fl^ (WT) mice. Original magnification is 20×. The symbols on the bar graphs are representing individual groups: circles-Rhot1^fl/fl^—Air; squares-Rhot1^fl/fl^—CS; upward triangles-Rhot1^CreCC10^—Air; downward triangles-Rhot1^CreCC10^—CS. Data are shown as mean ± SEM (*n* = 7 to 20 per group based on number of images per mouse group).

Lung sections from both WT (Rhot1^fl/fl^) and KO (Rhot1^CreCC10^) mice that were chronically exposed to CS for 4 months did not show significant airspace enlargement in compared to the air group (Figure 6).

## 4. Discussion

Chronic inflammation of the lungs is the hallmark of the pathogenesis of COPD, which is primarily caused by cigarette smoking. Cigarette smoke contains large amounts of toxic chemicals and free radicals that affect lung cells due to the deleterious nature of released oxidants and free radicals [27,28,29]. The dysfunction in mitochondria due to injury and morphological alterations are shown to be significantly driven by CS exposure to the lung cells, including airway and alveolar epithelial cells, fibroblasts, and airway smooth muscle cells [30,31,32]. However, the mechanism behind this cellular damage and inflammatory response and the role of Miro1 in mitochondrial dysfunction upon exposure to CS in lung epithelial cells is remained unclear. We have previously reported the association of Miro1 reduction with CS-induced inflammation in lung epithelial cells [1]. Therefore, based on our findings and other reported studies on the significant role of Miro1 in regulating the mitochondrial quality control system, we intended to study the severity of CS-induced lung inflammation in the absence of Miro1 in epithelial cells and its association with mitochondrial dysfunction.

In this study, the Rhot1^fl/fl^ (WT) and lung epithelial cell-specific Rhot1^CreCC10^ KO mice were exposed to CS for acute (3 days) or chronic duration (4 months), and a comparative study of inflammatory responses between the Rhot1^fl/fl^ (WT) and Rhot1^CreCC10^ (KO) animals was performed to determine the role of Rhot1/Miro1 in CS-induced lung inflammation and in epithelial integrity. The role of mitochondrial dysfunction in lung inflammatory diseases, such as COPD, is shown earlier [33,34]. The regulation of cellular events, such as the fission (fragmentation) and fusion (elongation) processes, allows the mitochondria to rapidly change their shape. Mitofusins (Mfns, membrane-anchored proteins: Mfn1 and 2) and OPA-1 (optic atrophy 1) are the major proteins that allow mitochondrial fusion, which is achieved by interacting with other proteins such as Pink1 in the mitochondrial membranes [35]. The association of Miro1-mediated mitochondrial dysregulation in the augmentation of inflammatory responses was evident in our study and appeared to be a result of an intensified increase in the lung infiltration of total inflammatory cells, macrophages, and neutrophils in the bronchoalveolar lavage (BAL) fluid in the Rhot1^CreCC10^ (KO) mice after acute and/or chronic exposures to CS. There were no significant changes that were observed in the CD4 and CD8 cell counts. However, the CD4 cells showed non-significant but decreasing trends both in Rhot1^fl/fl^ (WT) and Rhot1^CreCC10^ (KO) animals that were exposed to cigarette smoke. The decreasing trends in the number of CD4 cells indicates a trend towards a localized effect in the cell-mediated immune response due to acute CS exposure and suggests the possibility of compartmentalized immunosuppression. Moreover, a significant increase in the macrophages and neutrophils suggests an association of inflammatory cell influx with acute and/or chronic lung inflammation. This was associated with increased inflammatory mediators, such as MIP-1α, IL-6, MCP-1, KC, IL-1β, G-CSF, and IL-12p40 in discussion the Miro1^CreCC10^ KO group. Moreover, the exaggerated abundances in G-CSF, KC, MCP-1, and MIP1-α after chronic CS exposure in the Rhot1^CreCC10^ (KO) group indicate subsequent inflammatory cell infiltration occurrences as well as a role of Miro1 in inducing and intensifying these changes. Further, these findings substantiate that any prolonged accumulation of infiltrating cells, such as neutrophils and macrophages, act as prime events in the pathological contribution of cigarette/tobacco exposure to the lung [36,37].

The disruptions in the mitochondria refer to various events that occur at the cellular level in the mitochondria, such as morphological changes, alterations in metabolic activity, decreased membrane potential and altered mitochondrial superoxide levels, and intracellular Ca^2+^ flux [6,7]. This may alter the dynamic nature of the organelle. CS exposure causes an alteration in mitochondrial dysregulation by modifying its function and mitophagy, and resulting in ROS production [2,30], leading to apoptosis. The histopathological data of the lung sections after chronic CS-exposure in the Rhot1^CreCC10^ (KO) mice did not reveal any significant airspace enlargement compared to the air- and CS-exposed Rhot1^fl/fl^ (WT) mice. Nevertheless, other changes, such as the accumulation of pulmonary interstitial macrophages and signs of epithelial hyperplasia/dysplasia (columnar cell-dysplasia) in the airway region of the CS-exposed mouse lungs were observed, implicating the role of epithelial Miro1 in regulating epithelium integrity and that inflammatory cell influx is associated with epithelial damage. We observed the presence of foamy macrophages in the lung interstitium (macrophages undergone structural changes) in lung tissues after the 4 months of CS exposure. The changes were more intensified in the Rhot1^CreCC10^ (KO) mice. Interestingly, G-CSF and MIP-1α, which regulate the release and molecular characterization of granulocytes, were found to be increased in the Rhot1^CreCC10^ (KO) mice after 4 months of chronic CS exposure, which also supports the occurrence of epithelial hyperplasia/dysplasia, and that lung inflammatory cell influx is directly associated with epithelial injurious responses. The lung inflammation has an impact the airways, with smaller airways being more predominant at the early stages of COPD [7,35]. However, after 4 months of the CS exposure in these mice, it possible that the destruction and the enlargement processes may proceed both chronologically and simultaneously. Mechanical properties and airspace enlargement in lungs had no comparable changes in the Rhot1 group in either the air and CS groups, which was also the case in the WT and Rhot1^CreCC10^ (KO) groups. The relatively shorter duration, particulate amount/concentration of CS exposure, and the genetic nature of mice may explain the non-significance of the structural changes that were observed in these mice. It is possible that the longer duration of CS exposure may have induced airspace enlargement and alteration in the lung mechanical properties.

There are several studies that point to Miro1/Rhot1 having a role in the mitochondrial quality control mechanism [9,38,39,40,41]. Our findings strengthen the idea that Miro1 is a key player in exerting inflammatory responses due to CS exposure in the lungs, which is in part mediated by CS-induced mitochondrial dysfunction/mitophagy and mitochondrial quality control. This study was limited to the study of CS-induced acute and prolonged inflammatory effects between WT and Rhot1^CreCC10^ (KO) mice. Thus, these findings warrant further studies to specify the role of Miro1-mediated mitochondrial dysfunction in causing smoking-related lung effects. Nevertheless, the mechanism for the role of Miro1 that was identified here may provide insight into strategies that focus on pharmacologically restoring the role of mitochondrial quality control mechanisms to treat inflammatory lung conditions, such as COPD.

## 5. Conclusions

Epithelial Rhot/Miro1 ablation was shown to augment both acute- and chronic-induced inflammatory responses in lungs associated with cellular infiltration with alteration in the levels of pro-inflammatory cytokines and histopathological changes in mouse lungs. This suggests a possible role of Miro1 in the CS-induced progression and development of inflammatory responses, some of which lead to the development of chronic lung diseases.

Our findings provide insight for further studies to determine the role of Miro1 in regulating the mitochondrial quality control mechanisms induced by CS in the pathogenesis of COPD/emphysema. This may have implications for the development of strategies for the pharmacological manipulation of Miro1/mitochondrial quality control mechanisms in therapeutic strategies for lung diseases.

## Data Availability

All data are provided in this manuscript.

## Supplementary Materials

The following are available online at https://www.mdpi.com/article/10.3390/pathophysiology28040033/s1, Figure S1: Differential expression of cytokines in epithelial cell–specific Rhot1 deleted and WT mice (acute exposure), Figure S2: Differential expression of cytokines in epithelial cell–specific Rhot1 deleted and WT mice (chronic exposure).
