# Supplementary material for: Epithelial Ablation of Miro1/Rhot1 GTPase Augments Lung Inflammation by Cigarette Smoke
Source: Pathophysiology. 2021 Nov 26;28(4):501–12. doi: 10.3390/pathophysiology28040033 (PMC8830451; doi:10.3390/pathophysiology28040033)
Supplement: Supplementary file 1 [file pathophysiology-28-00033-s001.zip › pathophysiology-1407035-supplementary.pdf]

Supplementary Materials

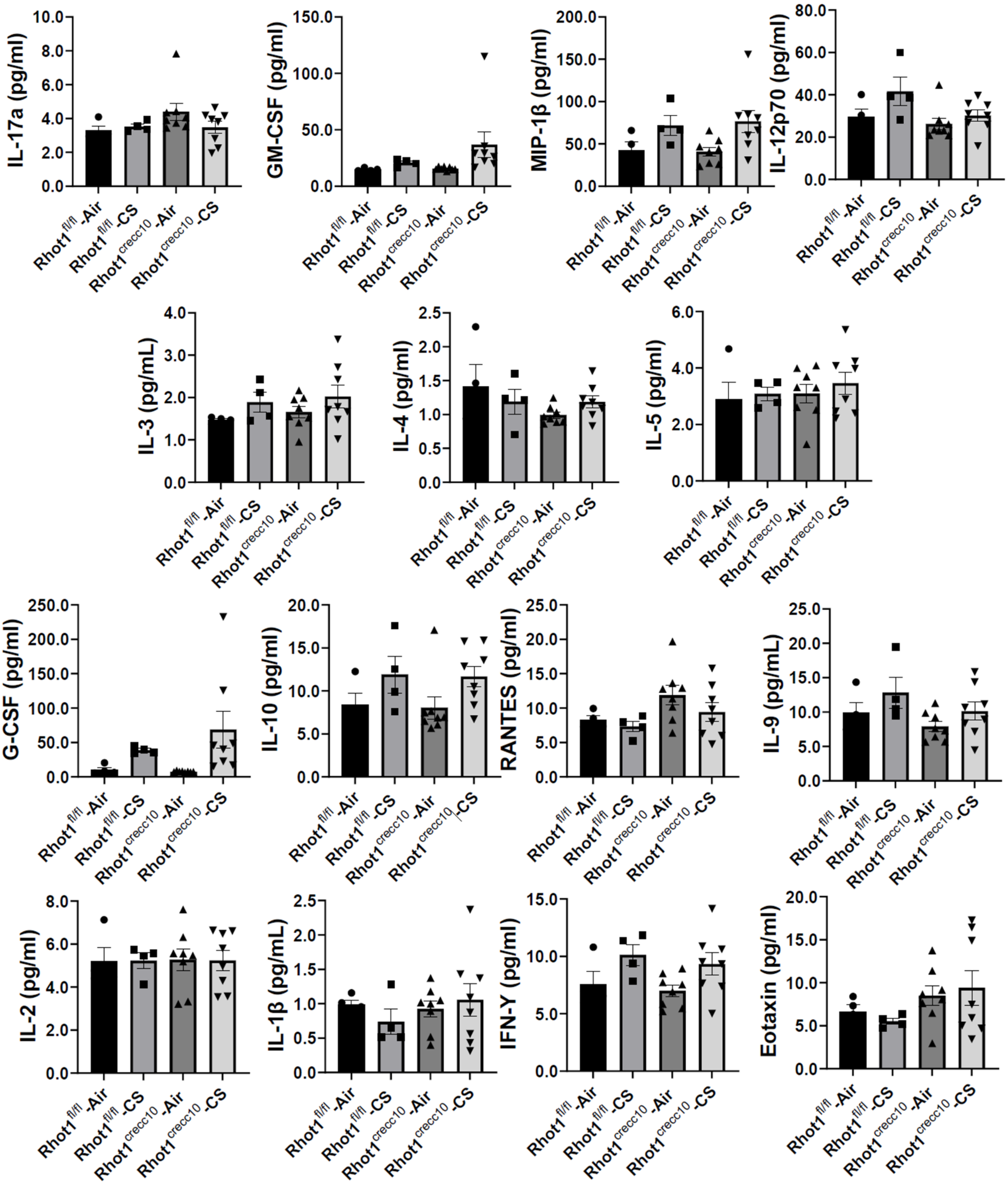

Figure S1. Differential expression of cytokines in epithelial cell–specific Rhot1 deleted and WT mice: Rhot1$^{Fl/Fl}$ (WT) and Rhot1^{CreCC10} (Rhot1 flp CreCC10$^{+/-}$ and Rhot1 flp CreCC10$^{+/+}$) mice were exposed to room air and CS (mainstream) for 3 days (acute exposure). Expression levels of pro-inflammatory and inflammatory mediators in BAL fluid room air and CS-exposed mice for 3 days was determined using Bio-Plex Pro 23-plex cytokine assay. Data are shown as mean ± SEM (n = 5 to 8 per group).

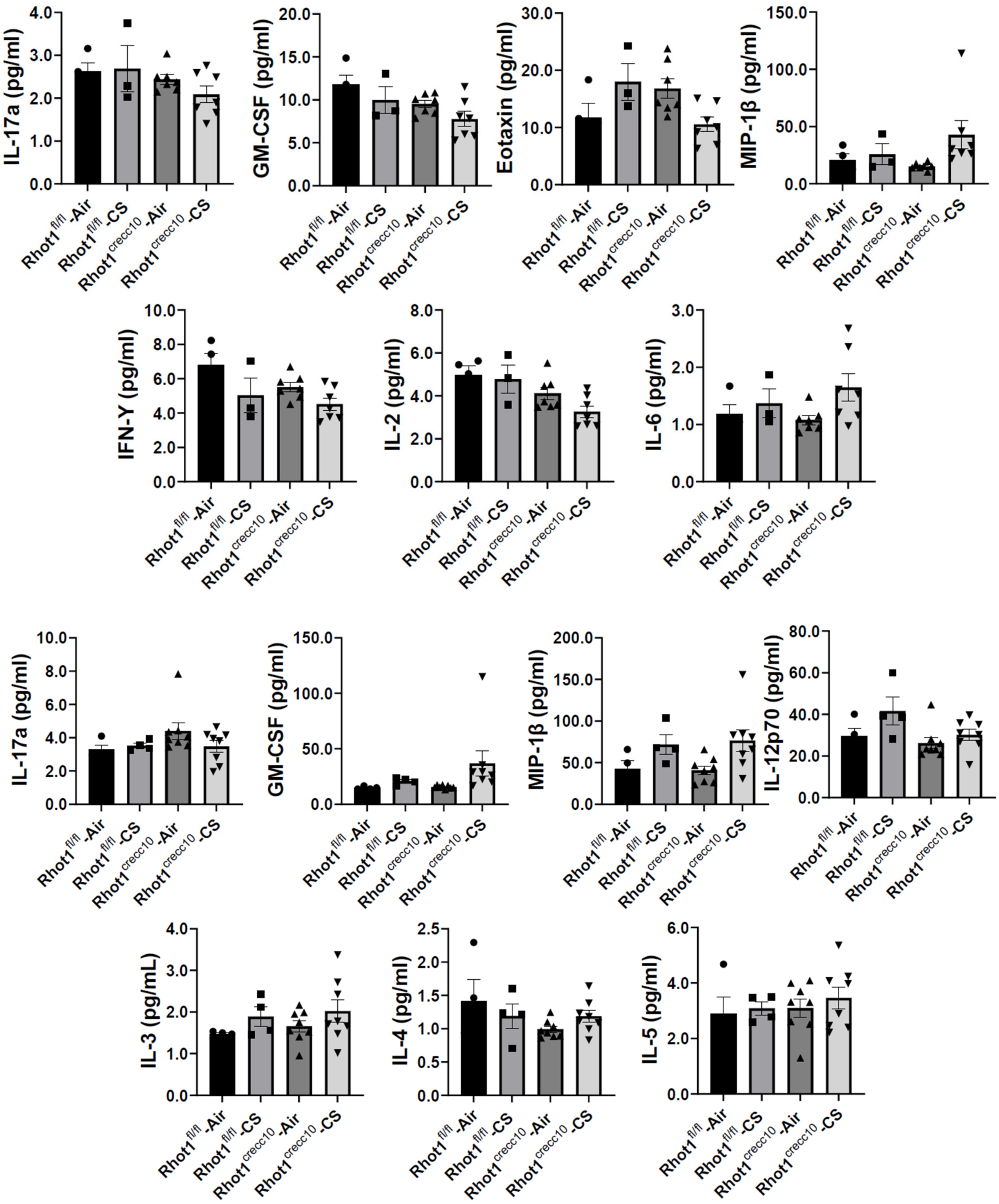

Figure S2. Differential expression of cytokines in epithelial cell–specific Rhot1 deleted and WT mice: $Rhot1^{Fl/Fl}$ (WT) and $Rhot1^{CreCC10}$ (Rhot1 flp $CreCC10^{+/-}$ and Rhot1 flp $CreCC10^{+/+}$) mice were exposed to room air and CS (mainstream) for 4 months (sub-chronic exposure). Expression levels of pro-inflammatory and inflammatory mediators in BAL fluid room air and CS-exposed mice for 4 months as determined using Bio-Plex Pro 23-plex cytokine assay. Data are shown as mean ± SEM (n = 3 to 7 per group).
